# Multi-Visceral Transplantation in a 21-Year-Old Man with Prior Pancreatoblastoma

**Published:** 2016-08-01

**Authors:** R. Girlanda, A. Pozzi, C. S. Matsumoto, T. M. Fishbein

**Affiliations:** 1 MedStar Georgetown Transplant Institute, Washington, DC, USA; 2 *San Raffaele Hospital, Vita-Salute San Raffaele University, Milan, Italy*

**Keywords:** Transplantation, Immunosuppression, Short bowel syndrome, Liver failure, Neoplasms, Neoplasm Metastasis

## Abstract

Organ transplantation in patients with prior malignancy increases the risk of tumor recurrence post-transplantation due to immunosuppression. Only two cases of liver transplantation have so far been reported in children with hepatic metastases from pancreatoblastoma, a rare malignant neoplasm originating from the epithelial exocrine cells of the pancreas. Herein, we describe a case of a successful multi-visceral transplant in a man with intestinal failure after surgical resection of pancreatoblastoma.

## CASE REPORT

**Figure 1:**
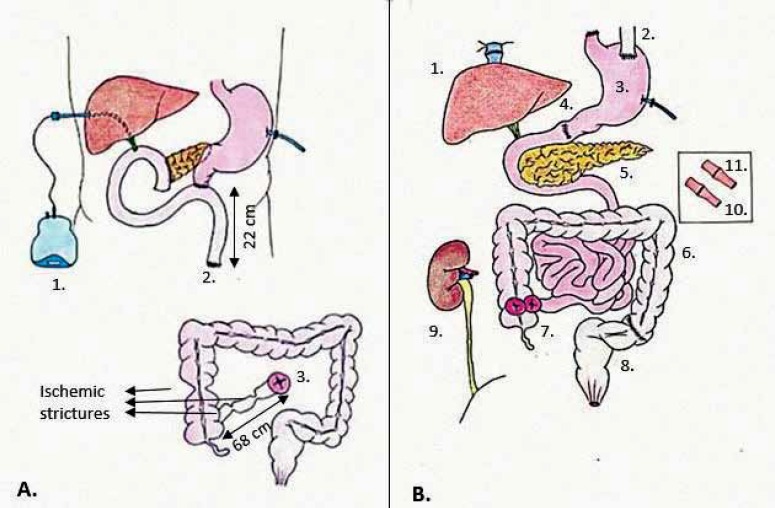
Representation of the patient’s anatomy before and after surgery. (A) 1. Percutaneous Transhepatic Cholangiography; 2. Stapled-off Jejunum; 3. Ileostomy with mucous fistula; and (B) 1. Piggyback liver transplant; 2. Native esophagus; 3. Stomach graft; 4. Piloroplasty; 5. Pancreas graft; 6. Colon graft; 7. Loop ileostomy; 8. Native sigmoid colon; 9. Kidney graft; 10. Donor celiac trunk-recipient celiac trunk anastomosis; 11. Donor superior mesenteric artery-recipient superior mesenteric artery anastomosis

A 17-year-old Arab male in good general health presented to his gastroenterologist with abdominal pain, weight loss, and diarrhea. The diagnostic work-up, including axial abdominal imaging, revealed a 9-cm mass in the body of the pancreas involving the superior mesenteric artery (SMA) and vein (SMV). The biopsy of the mass was consistent with pancreatoblastoma (PB). The patient received neo-adjuvant chemo-radiotherapy with partial response. Two years later, at another center, he underwent surgical resection with a pylorus-sparing pancreato-duodenectomy including resection of the involved SMV and SMA. The reconstruction of the SMA required the use of an autologous saphenous vein graft from the aorta. Histological examination of the resected mass confirmed PB measuring 5×4.5×4.5 cm with perineural and peripancreatic invasion and infiltration of 1/16 peripancreatic lymph nodes. The margins of the excised SMA were negative. The patient’s coagulation profile was significant for factor V Leiden mutation heterozygosity (R506Q) and lupus anticoagulant positivity. The post-operative course was complicated by mesenteric ischemia with marked leukocytosis, acidosis, and severe hemodynamic instability requiring inotropic support. On post-operative day 12, the patient required re-operation and underwent sub-total enterectomy for small intestine necrosis secondary to thrombosis of the saphenous vein graft. The length of the residual small intestine consisted of 22 cm of proximal jejunum (stapled off) and 68 cm of distal ileum (mucous fistula) in continuity with the colon (Fig 1). The foregut was decompressed via gastro-jejunostomy tube and the bilio-pancreatic tract was diverted with percutaneous trans-hepatic external drainage. Total parenteral nutrition was initiated and the patient was referred to our center for intestinal transplantation. At the time of referral, the patient’s serum total bilirubin level was 11.8 (direct 8.2) mg/dL; liver biopsy demonstrated cholestasis and stage II fibrosis, consistent with parenteral-nutrition-associated liver disease.

During the evaluation for candidacy to intestinal transplantation, attempts were made at resuming enteral nutrition via a feeding tube through the residual ileum. These attempts failed due to chronic stricturing of the ileum secondary to previous ischemic insults. In addition, ischemic strictures at the level of the ascending colon were detected on barium enema. An extensive malignancy workup, including whole-body nuclear scan revealed multiple hypermetabolic soft tissue foci within the abdomen around the aorta, in the supraclavicular and inguinal lymph nodes, and one hypermetabolic focus within the liver, suggestive of metastatic PB. Multiple attempts to characterize these lesions with CT-guided biopsy were inconclusive. Therefore, the patient underwent a staging laparotomy. In addition, the laparotomy was aimed to determine the viability of the residual small and large intestine for potential restoration of the gastrointestinal continuity and resumption of enteral nutrition. No macroscopic evidence of PB was found intra-operatively; biopsies taken from mesenteric and peri-aortic lymph nodes as well as the liver, were negative for malignancy. The residual proximal jejunum appeared viable and was decompressed with creation of a high jejunostomy. Unlike the jejunum, the distal ileum and the ascending colon appeared chronically strictured at multiple levels and were resected. As a result, the residual short gut was not reconstructable rendering the patient totally dependent on parenteral nutrition with no potential for intestinal rehabilitation. Having excluded, to the best of the available diagnostic accuracy, the presence of residual or recurrent PB and having completed the pre-transplantation evaluation, as per our protocol, the patient was added to the wait-list for multi-visceral transplantation. While on the wait-list, the patient suffered multiple catheter-related blood stream infections and repeated episodes of acute kidney injury requiring admission to the intensive care unit and ultimately developed end-stage kidney failure requiring hemodialysis. 

Fourteen months after listing, the patient underwent multi-visceral and kidney transplantation. The donor was a blood-type O 15-year-old female with weight of 61 kg, height 173 cm, and BMI of 20 kg/m^2^ who suffered brain death following a head trauma from motor vehicle accident. The donor was hemodynamically stable with no acidosis and normal liver and kidney function. The abdominal organs were free from injury. We recovered an *en-bloc *multi-visceral graft consisting of liver, stomach, duodenum, pancreas, small intestine, and large intestine up to the distal transverse colon. The left kidney was recovered from the same donor as well.

After completion of the recipient’s native enterectomy, hepatectomy and pancreatectomy, the multi-visceral graft was transplanted as previously described [1]; the kidney graft was transplanted intra-peritoneally to the right iliac fossa with standard technique (Fig 1). The arterial inflow to the multi-visceral graft consisted of two separate end-to-end anastomoses between the celiac trunk and the SMA of the recipient and of the graft, respectively (Fig 1). Due to the small abdominal domain, the abdomen was closed with biologic mesh in order to avoid increased intra-abdominal pressure. Induction immunosuppression consisted of anti-IL-2R antibodies; maintenance immunosuppression was with tacrolimus and corticosteroids. The post-transplantation monitoring with ileoscopy and biopsy was unremarkable with no rejection episodes and normal kidney graft function. The post-transplantation course was uneventful with excellent graft function and the patient was discharged home on post-operative day 27. At five years post-transplantation, the patient is disease-free with normal graft function on low dose maintenance immunosuppression.

## DISCUSSION

PB is a rare epithelial neoplasm of the exocrine pancreas, reported for the first time in the surgical literature by Becker in 1957; it is originally termed “infantile pancreatic carcinoma” [2]. The term “pancreatoblastoma” was first proposed by Horie, *et al*, in 1977 to refer to a pancreatic tumor with histological resemblance to fetal pancreatic tissue at approximately seven weeks of gestation; “pancreatoblastoma” has since become the accepted term to describe this tumor, histologically characterized by relatively dense cellularity, a nesting growth pattern, acinar differentiation and the presence of characteristic “squamoid corpuscles” [3]. Since then, approximately 200 cases of PB have been reported in children and about 40 in adult patients. In the adult population, the tumor predominantly affects Asian males [4]. The prognosis of PB in adults is significantly worse than in children, with metastases occurring in 26% of cases, mainly to the lymph nodes and the liver [5]. Our knowledge of the clinical course and treatment of PB is limited, given the small number of cases. Therefore, there remains a poor understanding of the natural history of the tumor, and more importantly, a lack of a standardized diagnostic and therapeutic approach. 

According to the literature, radical surgical resection is the treatment of choice in patients with localized disease, offering the only chance of cure with 75%–80% event-free survival after initial or delayed resection. Even in patients with liver metastases, there is consensus that aggressive surgical management should be pursued [6]. Although the role of adjuvant chemotherapy or radiotherapy is still debated, due to the small number of patients, neoadjuvant chemotherapy is indicated in large tumors involving adjacent major blood vessels or other organs and in metastatic disease. Chemotherapy may be the only option in patients with unresectable disease [7]. 

The case that we describe in here, involved the transplantation of a multi-visceral graft in a patient with intestinal failure and life-threatening complications from parenteral nutrition following the treatment for PB. In our patient, transplantation was the only option to restore enteral autonomy and allow discontinuation of parenteral nutrition. Our case highlights the challenges of ruling-out residual malignant disease at the time of transplantation and of balancing adequate immunosuppression levels post-transplantation in order to minimize the risk of tumor recurrence.

Only two cases of liver transplantation have so far been reported in children with hepatic metastases from PB [8, 9]. To our knowledge, there are no previous case reports of patients with history of PB undergoing multi-visceral transplantation. Typically, multi-visceral transplant recipients are treated with heavy immunosuppression regimens to prevent rejection, thereby, significantly increasing the risk of tumor recurrence post-transplantation. Our patient currently remains free from rejection episodes and from recurrence of PB five years after transplantation. We believe that a careful patient selection, a strict multi-disciplinary monitoring and close follow-up are critical in high risk transplant recipients such as the patient presented here.

## Notes

**FINANCIAL SUPPORT:** None.
